# Smart and inclusive tourism in Bukhara: towards accessibility in heritage city

**DOI:** 10.3389/fspor.2025.1624663

**Published:** 2025-11-17

**Authors:** Yulduz Dushanova, Shafoat Kadirova, Mohinur Kurbanova, Dilora Khaydarova

**Affiliations:** 1 Tourism and Hotel Management Department, Faculty of Economics and Tourism, Bukhara State University, Bukhara, Uzbekistan; 2 Department of Business Organization, Universitat Politècnica de València, Valencia, Spain; 3 Russian State Tourism and Service University, Moscow, Russia

**Keywords:** smart tourism, accessibility, inclusive tourism, heritage destinations, universal design, Bukhara, sustainable tourism, mega-events

## Introduction

Bukhara's embrace of “smart tourism”—integrating digital technology into visitor experiences (1) brings both opportunities and responsibilities. Globally, mobile apps, augmented reality guides, and data analytics are enriching how tourists engage with cultural sites. Uzbekistan's tourism strategy prioritizes digital transformation, with cities like Tashkent and Samarkand piloting e-ticketing and interactive maps. Bukhara has begun to follow suit: QR codes at dozens of historic monuments offer information in multiple languages, and plans have been floated for virtual tours and 3D models of heritage sites. These initiatives enhance visitor engagement and help manage tourist flows. However, a truly “smart” destination must also be inclusive. International best practices frame accessibility as a core component of smart tourism—the European Capital of Smart Tourism program, for instance, requires attention to accessibility alongside innovation (2). A heritage city cannot be considered successfully “smart” if wheelchair users, elderly visitors, or those with vision or hearing impairments cannot comfortably enjoy its attractions. Smart solutions should therefore **augment** accessibility, not create new barriers. In Bukhara's context, technology should be harnessed to improve access—for example, using mobile apps to guide mobility-impaired visitors via barrier-free routes or providing virtual visits to spaces that remain physically challenging—marrying modern tech with universal design principles.

**Figure 1:**
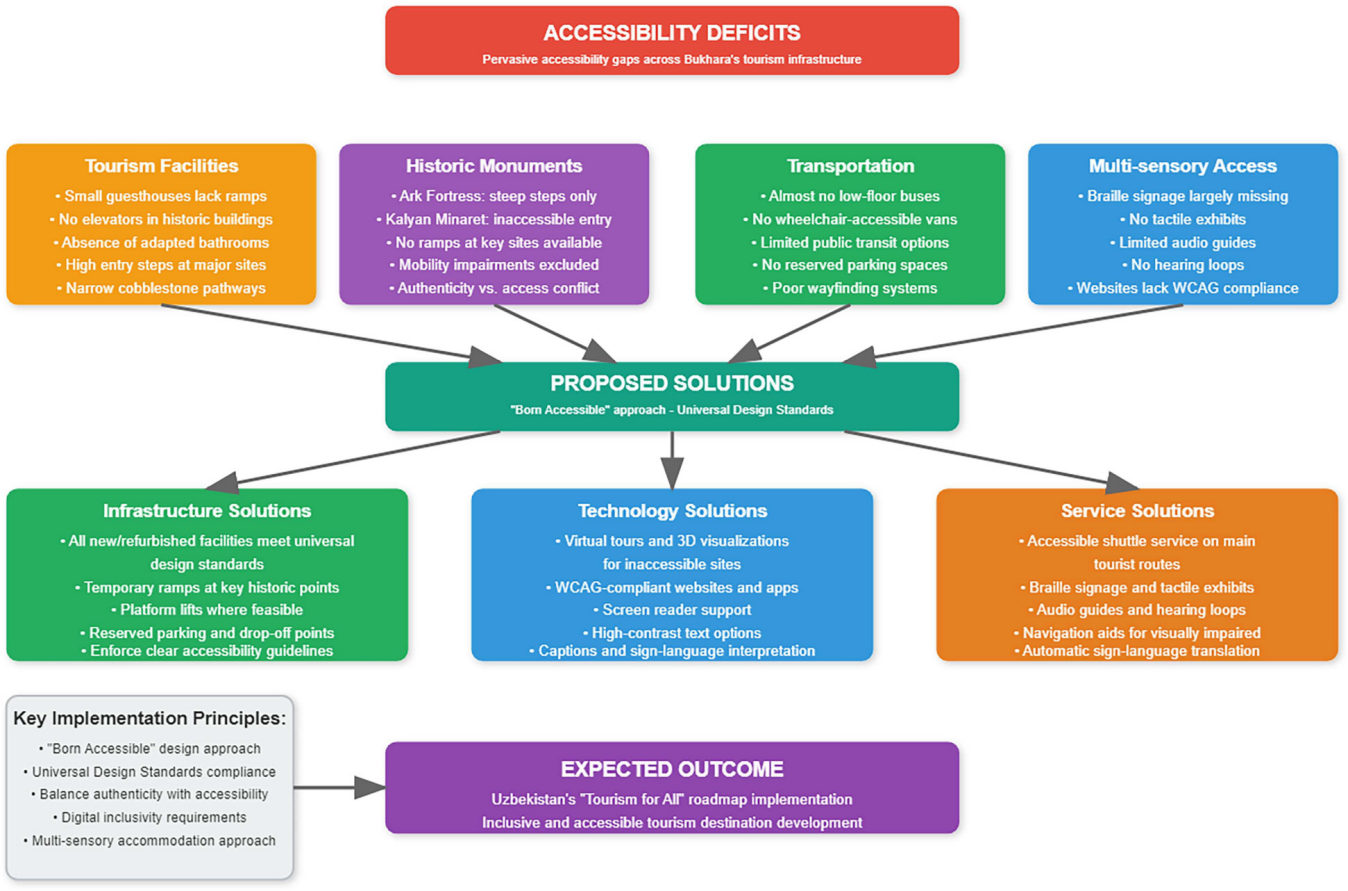
Bukhara's smart tourism accessibility: problems and solutions.

Many of Bukhara's tourism facilities still lack basic accessibility features. Small guesthouse hotels in historic buildings often have no ramps, elevators, or adapted bathrooms; even major sites have high entry steps and narrow cobblestone pathways that pose obstacles for wheelchair users. A recent local survey found pervasive accessibility deficits across Bukhara's hotels, public areas, and museums (3). Going forward, all new or refurbished tourism infrastructure should be *born accessible*—designed or upgraded to meet universal design standards—and local authorities must enforce clear accessibility guidelines (4). Figure 1 summarizes the main accessibility deficits identified across Bukhara's tourism infrastructure—including in facilities, monuments, transport, and digital access—and presents proposed solutions grounded in universal design standards. It outlines specific infrastructure, technology, and service improvements aimed at making Bukhara an inclusive heritage destination aligned with Uzbekistan's “Tourism for All” roadmap.

Historic monuments pose similar challenges. Iconic sites like the Ark Fortress and Kalyan Minaret are only reachable via steep steps, with no ramps or lifts for those with mobility impairments. While preserving authenticity is important, it can be balanced with access: for example, temporary ramps or platform lifts could be installed at key points, and where physical access is impossible, virtual tours or 3D visualizations can offer an alternative experience (4). Transportation and wayfinding are also problematic in Bukhara. The city has almost no low-floor buses or wheelchair-accessible vans, so travelers with disabilities have limited public transit options. Introducing an accessible shuttle service on main tourist routes, along with reserved parking or drop-off points at major sites, would greatly improve mobility. In addition, accommodations for blind or deaf visitors are scarce—Braille signage, tactile exhibits, audio guides, and hearing loops are largely missing—so investing in these multi-sensory aids would help make cultural sites usable by all. Bukhara's push for e-tourism also needs to be inclusive. The city's websites and apps should comply with accessibility standards (e.g., Web Content Accessibility Guidelines) so that information is perceivable and usable by people with visual, hearing, or cognitive disabilities. For instance, interfaces should support screen readers, offer high-contrast text options, and include captions or sign-language interpretation for multimedia content. Uzbekistan's national “Tourism for All” roadmap calls for such features, including navigation aids for visually impaired users and automatic sign-language translation in tourism apps (4). By adopting these digital accessibility practices, Bukhara can ensure that its smart tourism tools serve all users.

Bukhara is not alone in grappling with how to make an ancient city more accessible. Heritage cities such as Kraków (Poland) and Konya (Turkey) have demonstrated that improving accessibility need not come at the expense of authenticity. Both cities conducted audits of their cultural sites and found strong demand from disabled and senior visitors, then implemented practical adaptations: installing ramps or elevators in historic buildings where feasible, providing information in Braille and sign language, ensuring accessible toilets and parking, and training staff in disability awareness. In Konya's famous Mevlana Museum, for example, a small elevator was added unobtrusively to allow wheelchair access without altering the historic façade. These efforts show that architectural heritage can be preserved while making sites more user-friendly for all. Meanwhile, Europe's “smart tourism capitals” such as Helsinki and Málaga have leveraged technology to further inclusion—for instance, mobile apps that map barrier-free routes and real-time transit accessibility, or virtual reality tours of attractions that wheelchair users cannot access in person. A recent analysis found that digital accessibility features (e.g., user-friendly websites, multilingual chatbots, audio guides with descriptions) were integral to the success of these leading smart destinations (5). Innovation must serve *all* users, underscoring that accessibility is a pillar of smart tourism (6).

Apart from being socially just, accessible tourism is also economically *smart*. Travelers with disabilities (along with their companions) form a sizeable market that tends to spend more and stay longer if appropriate facilities are in place. Destinations that invest in ramps, lifts, and other adaptations can attract this underserved segment and gain a reputation as welcoming cities, translating into higher visitor numbers and revenue. For example, world-class heritage cities like Barcelona report that improving physical accessibility has enhanced overall tourist satisfaction and competitiveness. In Uzbekistan, policymakers note that inclusive tourism can significantly expand the visitor economy (4). Indeed, an official decree in Uzbekistan (President's Resolution PQ-20, Jan 2024) explicitly calls for developing “*barrier-free tourism”* infrastructure nationwide, reflecting a growing recognition that accessibility and innovation go hand in hand. The UN World Tourism Organization (UNWTO) has produced extensive guidelines on accessible tourism, emphasizing that everyone should have equal access to tourism experiences (7, 8). Recently, UNWTO, in partnership with disability organizations, issued new recommendations based on the international standard ISO 21902:2021 *Tourism for All*, targeting cultural tourism stakeholders who wish to make their offerings more accessible. This global momentum provides Bukhara an opportunity to align with international standards and best practices. Adopting formal guidelines like ISO 21902 (Tourism for All—Requirements and Guidelines) in city regulations would ensure that accessibility is systematically integrated into tourism planning and heritage management. By benchmarking against such standards, Bukhara can evaluate its progress and identify gaps in both physical and digital accessibility.

## Comparative perspective: Samarkand and Khiva

Within Uzbekistan, other historic cities offer useful comparisons. **Samarkand**, a larger Silk Road city and UNESCO World Heritage site, has invested in modern tourism infrastructure and recently hosted major international events. As a result, Samarkand has made some strides in accessibility, at least in its main tourist areas. For instance, observers note that in the famous Registan Square, ramps have been installed at various points (albeit some are steep or slippery due to marble), and overall “*Samarkand is much more… geared up for wheelchair users”* compared to other cities. This is likely because Samarkand attracts a high volume of international tourists and has received government and donor attention for upgrades. The city introduced electric buses and improved wayfinding for visitors ahead of hosting the 2022 SCO summit, steps which also benefited those with mobility issues. Nonetheless, many historical sites in Samarkand (such as Shah-i-Zinda or certain madrasahs) remain challenging to access due to stairs and uneven surfaces, indicating that further work is needed to achieve full inclusion. In contrast, **Khiva**—a smaller walled museum-city—retains an almost untouched medieval urban fabric. Its narrow mudbrick alleys and high fortress walls present inherent accessibility obstacles. Basic improvements (like wooden ramps over thresholds) have been added at some entrances, but the options for major physical alterations are limited by Khiva's desire to preserve authenticity. Still, even in Khiva, tour operators have begun to identify which attractions can be accessed by wheelchair and to offer adaptive services. One experienced traveler remarked that finding accessible hotels in the main tourist cities of Uzbekistan (Tashkent, Samarkand, Bukhara, and Khiva) is “*not a big problem”* if one chooses newer or higher-end accommodations. The bigger challenges are navigating historic streets and finding accessible public conveniences (in the words of the traveler, “*the main problem is to find toilets during the day”*). These comparisons suggest that while Bukhara is not uniquely disadvantaged, it also has not yet taken the lead on accessibility. By learning from Samarkand's incremental improvements (e.g., installing ramps at key sites) and acknowledging Khiva's constraints (and creative small-scale solutions), Bukhara can chart a course that suits its own scale and heritage context. In short, all three cities need to progress toward inclusive tourism, but Bukhara has the chance to differentiate itself by proactively becoming Uzbekistan's model “barrier-free” heritage city. The national government's current focus on inclusive tourism development, and programs like “Inclusive Journey” which organized thousands of trips for people with disabilities in 2023, create a supportive environment for Bukhara to push forward on this agenda (9).

## Recommendations for a smart & accessible Bukhara

To transform Bukhara into a truly smart *and* inclusive tourist destination, a multi-pronged strategy is required. First, city tourism authorities should commission a detailed accessibility audit of Bukhara's entire tourism value chain—from transit hubs and sidewalks to hotels, restaurants, and cultural attractions. This audit, ideally conducted with input from disability advocacy groups, will pinpoint specific barriers (e.g., missing ramps, lack of Braille signage, non-accessible restrooms) and help prioritize fixes. Establishing a quantified baseline in this way allows measurable targets to be set for improvement. It can also identify “quick wins” (such as installing portable ramps at a few key choke points) vs. longer-term infrastructure projects (like adding elevators in museums or creating accessible paths in the old city).

Second, embed universal design principles in all tourism development. New tourism facilities—whether a small guesthouse or a large museum—should not be approved unless they include accessibility features by design (for example, ramps, lifts, wide doorways, and adapted restrooms). Local building codes and heritage restoration guidelines need to incorporate accessibility criteria, aligned with international standards (13). Where strict preservation rules make physical modifications difficult, creative solutions should be sought: removable or invisible ramps, stair-climber devices, or digital alternatives (like virtual tours) to complement the on-site experience. Notably, the UNESCO World Heritage status of Bukhara permits adaptive measures that do not permanently alter the fabric of monuments, as long as they are reversible and respect authenticity (10). City officials can consult examples from European historic centers that successfully introduced wheelchair routes and tactile exhibits without compromising Outstanding Universal Value.

Third, orient Bukhara's digital initiatives toward accessibility. The city's nascent smart tourism apps and websites should comply with recognized digital accessibility guidelines (WCAG 2.1). For instance, the official tourism website and any mobile apps should support screen readers for blind users, provide high-contrast and text-resize options for visually impaired users, and include captions or sign-language inserts in video content for deaf users. Given that a 2023 study found none of the official websites of Europe's top smart-tourism cities to be fully compliant with accessibility criteria, Bukhara should strive to surpass the status quo by making its digital platforms *born accessible*. A simple step would be conducting an accessibility audit of existing websites (using tools that check for screen-reader compatibility, alt-text on images, etc.) and then transparently improving any shortcomings. Additionally, Bukhara could develop or adapt a mobile app for accessible tourism—for example, an app that highlights wheelchair-friendly routes through the Old City, lists accessible hotels/restaurants, provides audio descriptions of sites, and offers navigation assistance for visitors with visual impairments (11). Cities like Málaga and Helsinki have pioneered such apps mapping barrier-free routes, and Bukhara could partner with tech firms or universities to create a localized version. This would exemplify smart tourism innovation serving an inclusive purpose.

Fourth, invest in training and stakeholder engagement. Regular training should be provided for staff across the tourism sector—tour guides, museum docents, hotel and restaurant employees, transportation operators—focusing on disability awareness and inclusive customer service. Training modules can cover appropriate etiquette (e.g., how to offer assistance to a wheelchair user without patronizing), basic sign language greetings, and operation of any assistive equipment. By sensitizing front-line personnel and managers, the destination can ensure that visitors with disabilities receive respectful and effective service. Over time, this builds a local culture of hospitality that is confident in welcoming all tourists. The city could establish an ongoing advisory council on inclusive tourism, involving representatives from disability organizations, tourism businesses, and local government. This council would provide feedback on plans and monitor progress, helping maintain momentum and accountability.

**Figure 2:**
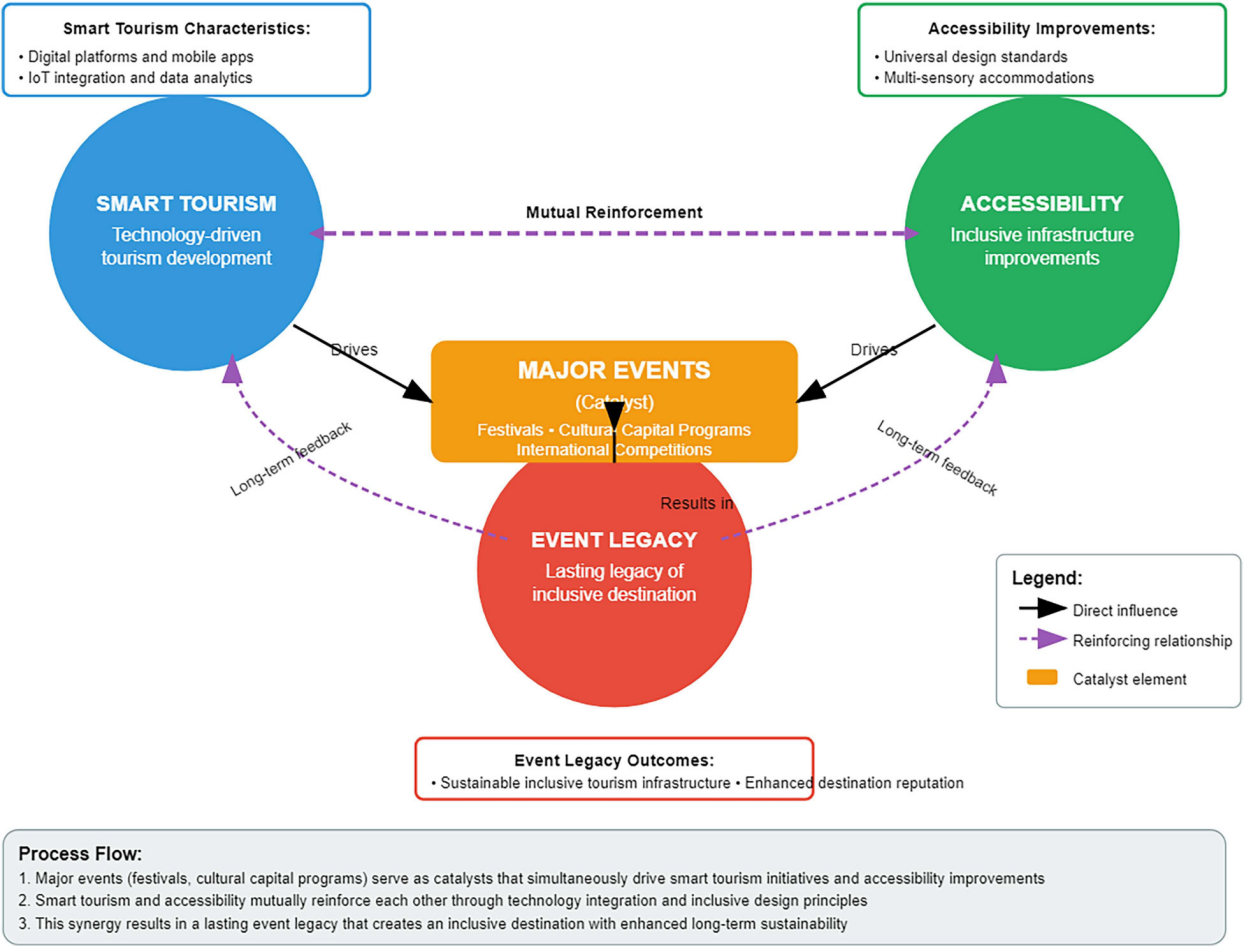
Conceptual relationship between smart tourism, accessibility, and event legacy. Major events (such as festivals or cultural capital programs) serve as catalysts that drive smart tourism initiatives and accessibility improvements, resulting in a lasting legacy of an inclusive destination.

Fifth, leverage major events as catalysts for accessibility. Large-scale events can spur cities to accelerate improvements and leave positive legacies. Bukhara has an upcoming opportunity in this regard: the city has plans to host a “Silk Road Expo” in the near future, and it regularly holds the annual Silk and Spices Festival which draws thousands of visitors. We recommend adopting an “accessibility by design” policy for all such events from the start. For any festival, cultural capital program, or international conference in Bukhara, organizers should include an accessibility plan with dedicated budget—for example, installing temporary ramps, lifts, or accessible portable toilets at event venues. These features can often be made permanent after the event. In fact, being named the Islamic Culture Capital in 2020 (12) already pushed Bukhara to consider infrastructure upgrades for an influx of international visitors; those efforts should be revisited and built upon. Any future bid to host a prestigious tourism forum or sports event could be tied to concrete accessibility targets—e.g., “*by the time of the 2025 Silk Road Summit, all major historic sites in Bukhara will have at least one accessible entrance and restroom.”* This approach follows the example of Olympic host cities like London (2012) and Tokyo (2020), which invested heavily in accessible infrastructure to meet event requirements, leaving a legacy of improved mobility for residents and visitors. While Bukhara may not host an Olympics, the principle stands: tying accessibility projects to the prestige and deadlines of a big event can galvanize funding and political will. Figure 2 below illustrates the reinforcing relationship between smart tourism, accessibility, and event legacy: major events act as a catalyst linking technology-driven tourism development with long-term inclusive infrastructure.

Finally, **promote and market Bukhara's inclusivity** once improvements are in place. Travelers with disabilities often rely on detailed information to plan their trips. Bukhara's tourism board should publicize any new accessibility features—for example, clearly label which attractions are wheelchair-accessible, create an “Accessible Bukhara” brochure or webpage, and work with specialized travel agencies and online forums to reach the disability travel community. Success stories of disabled visitors enjoying Bukhara can be shared to counter outdated perceptions. As one Uzbek disability advocate puts it, “*I want the world to become barrier-free… so that we can speak freely, express our ideas, and succeed”* (14). Showcasing Bukhara as a place that welcomes **all** guests will not only attract more visitors with special needs, but also appeal to the growing segment of tourists who value socially responsible destinations. In essence, inclusivity can be a selling point that distinguishes Bukhara in the Silk Road tourism circuit.

## Conclusion

Bukhara's journey toward smart tourism is a chance to set a new benchmark for historic cities by integrating digital innovation with inclusivity. The concept of a “smart and inclusive” Bukhara means that technology and tradition work hand in hand to make the city's rich heritage accessible to *all* visitors. Our examination highlighted notable gaps in physical and digital accessibility today, but it also identified practical pathways—drawn from international examples and upcoming opportunities—to bridge those gaps. By proactively implementing the recommendations above, Bukhara can transform its tourism landscape to ensure no visitor is left behind. This is not about gadgets for gadgets’ sake; it is about using every available tool, from a simple ramp to a sophisticated app, to open Bukhara's cultural treasures to a wider audience. Imagine an elderly tourist navigating the Old City with an intuitive audio guide, a wheelchair user accessing the Ark Fortress via a gentle ramp and enjoying a folk performance from a reserved viewing area, a deaf visitor engaging with museum exhibits through sign-language video guides, and a blind visitor exploring a 3D tactile model of a madrasa accompanied by audio description. These scenarios—once difficult to imagine—can become reality. They illustrate “tourism for all” in action. Making Bukhara more accessible and inclusive is ultimately an investment in the city's own legacy. Just as its architectural marvels have endured for centuries, the accessibility enhancements made today will enable Bukhara to remain a vibrant, welcoming destination for generations to come. Embracing this vision will position Bukhara as a pioneer of equitable, sustainable tourism in Central Asia, honoring the Silk Road spirit of hospitality and exchange.
